# “IT’S YOU”: CAREGIVER AND CLINICIAN PERSPECTIVES ON LUCIDITY IN PEOPLE LIVING WITH DEMENTIA

**DOI:** 10.1093/geroni/igae098.1551

**Published:** 2024-12-31

**Authors:** Andrea Gilmore-Bykovskyi, Clark Benson, Jess Fehland, Kim Mueller, Laura Block

**Affiliations:** University of Wisconsin Madison, Madison, Wisconsin, United States; University of Wisconsin School of Medicine and Public Health - Madison, WI, Madison, Wisconsin, United States; University of Wisconsin Madison, Madison, Wisconsin, United States; University of Wisconsin Madison, Madison, Wisconsin, United States; University of Wisconsin-Madison, Madison, Wisconsin, United States

## Abstract

Episodes of lucidity (ELs), or a transient return of abilities believed to be lost in people living with dementia, are a growing area of interest. These events hold important implications for care, caregiving, and our understanding of underlying etiologies. Research on ELs is largely limited to retrospective reports. The perspectives of professional and family caregivers on ELs and research approaches can inform efforts to define and study lucidity. The present study examined family caregiver and hospice clinician experiences with and perspectives on ELs in people living with dementia and observational approaches to studying these events. This exploratory, descriptive qualitative study employed semi-structured interviews (N=20 caregivers, N=6 clinicians). Data were analyzed using Rapid Identification of Themes and subsequent duplicate review of interview data to enhance trustworthiness. Most participants readily recalled events they perceived as ELs, describing a transient return of abilities they felt was significant and/or meaningful. Defining features, interpretations, and the perceived impact of ELs varied, although ELs were commonly conceptualized as a manifestation of self. Caregivers described extensive efforts to detect patterns and supportive social conditions for ELs. Participants supported use of audiovisual observation to study ELs and provided recommendations for privacy, workflow, and caregiver engagement. Interpretations of ELs are heterogeneous, and recognition of these events may necessitate close familiarity with the person living with dementia. Participants endorse observational approaches and integration of caregivers in this research.

